# Assessment of early macular microangiopathy in subjects with prediabetes using optical coherence tomography angiography and fundus photography

**DOI:** 10.1007/s00592-023-02167-z

**Published:** 2023-09-09

**Authors:** Shereen El Sawy, Mirrette Bekhit, Alaa Abdelhamid, Sohair Esmat, Hala Ashraf, Mervat Naguib

**Affiliations:** 1 Internal Medicine Department, Faculty of Medicine, Kasr Al-Ainy Hospital, Cairo University, 41 Manial Street, Cairo, 11451 Egypt; 2 Kasr Al-Ainy Vascular Laboratory, Cairo University, Cairo, Egypt; 3 Ophthalmology Department, Faculty of Medicine, Kasr Al-Ainy Hospital, Cairo University, Cairo, Egypt; 4 Clinical and Chemical Pathology Department, Faculty of Medicine, Kasr Al-Ainy Hospital, Cairo University, Cairo, Egypt

**Keywords:** Prediabetes, Retinopathy, Optical coherence tomography angiography, Vascular density, Prediabetes

## Abstract

**Aims:**

Early detection of retinal microangiopathy in patients with prediabetes may reduce diabetic retinopathy complications. The aim of this study was to assess early macular vascular changes in prediabetics before development of over diabetes using OCTA and fundus photography.

**Methods:**

In this cross-sectional study, 66 prediabetic individuals and 66 normal controls underwent clinical, laboratory, and fundus photography evaluation followed by OCTA macular imaging to examine for the foveal avascular zone, and area of capillary non-perfusion, thickness, disorganization of vessels, and vessel density perfusion percentage of superficial capillary plexus and deep capillary plexus.

**Results:**

Retinal microangiopathy was detected in 36.4% of prediabetics by OCTA and only in 10.6% by fundus photography. None of clinical or laboratory parameters had significant association with DR. Area of capillary non-perfusion and disorganization of SCP were detected in 53.8% and 56.8%, respectively, in prediabetics. VDP of SCP and DCP of whole image, parafoveal, and perifoveal areas was significantly lower in prediabetes group compared to normal control. VDP of DCP of perifoveal area (*β* coefficient: − 0.10, OR: 0.91, 95% CI: 0.86–0.96, *P* < 0.001) and disorganization of DCP (*β* coefficient: 1.93, OR: 6.89, 95% CI: 2.5–18.8, *P* < 0.001) were significant predictors of DR in prediabetics. There was no difference in FAZ in prediabetics with and without retinopathy.

**Conclusions:**

OCTA could detect early retinal vascular changes during the prediabetic state before developing diabetes. VDP was significantly reduced in prediabetic patients. Furthermore, VDP of DCP of perifoveal area and disorganization of DCP were the most important predictors of retinopathy in prediabetic patients.

## Introduction

Diabetes mellitus (DM), a major health problem, is preceded by a prediabetic state. Prediabetes is characterized by elevated glucose levels above normal levels and below diabetic ranges. Diagnostic levels of prediabetes include fasting glucose concentrations between 100 and 125 mg/dL and/or abnormal glucose tolerance between 140 and 199 mg/dL and/or glycated hemoglobin (HbA1c) between 5.7 and 6.4% [1].

Diabetic retinopathy (DR) is one of the main microvascular complications of DM. DR is believed to be a major cause of vision loss among middle-aged adults around the globe [2]. Some data suggest that microangiopathy of DM could start early during the prediabetes stage by the same picture and through the same pathogenic mechanisms including activation of the polyol pathway, advanced glycosylation, protein kinase C stimulation, and hexosamine pathway. Stimulation of the previous four pathways leads to a cascade of inflammatory response, fibrous disposition, damage to the blood vessels, and production of free radicals ending in tissue damage [3].

Early diagnosis of diabetic retinopathy is the best strategy to prevent or delay vision loss. Color fundus photography together with ophthalmoscopy represents the gold standard to identify and stage DR [4]. Fluorescein angiography is believed to be a prime tool widely used to evaluate retinal microvasculature but not preferable in patients who suffer mild DR or in cases without visible DR, besides the possible adverse effects of the dye injection [5]. Credit to technological development, optical coherence tomography angiography (OCTA) offers a novel imaging tool to visualize the microvascular changes that occur on the level of capillaries [6].

OCTA is rapid, is noninvasive, and does not need venipuncture or dye. It has a promising role in detecting diabetic macular ischemia and edema which are the main causes of blindness in DR [7]. OCTA provides highly detailed photographs focusing on the macular vasculature, as it quantitatively measures the region free of any capillaries named as foveal avascular zone (FAZ) [7]. Also, OCTA enables us to study both quantitatively and morphologically the retinal blood vessels without using any contrast [8]. OCTA is useful in the assessment of central and peripheral retinas [9].

Detection of vascular changes on OCTA in DR was assessed in previous studies, including microaneurysms, capillary non-perfused zones, FAZ, and neovascularization [10–12]. Some studies have evaluated FAZ area and macular vascular density in patients with diabetes [13, 14]. However, few data are available on the role of OCTA in the prediabetes stage [15]. Since diabetic microangiopathy could develop before overt diabetes is diagnosed, more research is needed to detect prevalence and extent of retinal microvascular affection in prediabetes. The aim of the current study was first to evaluate macular vascular findings using OCTA and fundus photography in a group of subjects with prediabetes compared to normal healthy controls and second to assess macular vascular changes in prediabetic eyes with DR compared to those without DR.

## Materials and methods

### Study design

This was a cross-sectional study.

### Study population

The subjects of this study were recruited from those who attended Internal Medicine and Diabetes, Endocrinology, and Nutrition outpatient clinics, Kasr Al-Ainy Hospital, Cairo University, from December 2021 to September 2022. Sixty-six persons with prediabetes represented the case group, and 66 healthy persons with normoglycemia represented the control group. Participants of the case group were fulfilling the criteria of prediabetes according to the ADA 2020 guidelines for prediabetes diagnosis [16]. Patients who had anemia, renal failure, liver cirrhosis, hypertension, history of smoking, previous ocular surgeries, glaucoma, and eye media opacities were excluded from the study.

### Clinical measurements and laboratory data

All participants were subjected to thorough medical evaluation including; age, gender, blood pressure measurement, weight, and height measurement to calculate the body mass index (BMI). The following laboratory tests were performed: fasting plasma glucose (FBG), two-hour postprandial glucose (2 h PPG), glycated hemoglobin (HbA1c), creatinine, and urinary albumin–creatinine ratio (ACR).

### Ocular examination

All participants were referred to the Ophthalmology Department, Diagnostic Laser Unit, Faculty of Medicine, Cairo University, where images of right and left eyes were obtained from each participant by color fundus photography and then by OCTA.

#### Color fundus photography

Macular and paramacular areas were examined through dilated pupils. Color fundus photographs were taken using a fundus camera Topcon TRC-50D for each eye. The presence of microaneurysms, intraretinal hemorrhages, hard exudates, cotton-wool spots, venous beading, intraretinal microvascular abnormalities (IRMA), and new vessels at the optic disk or elsewhere were recorded. Diagnosis of DR was based on the proposed international clinical DR and diabetic macular edema disease severity scales [17].

#### Optical coherence tomography angiography

OCTA images were obtained using the RTVue XR Avanti (AngioVue; Optovue Inc, Fremont, California, USA) machine. Scanning for each eye whiles the patient sits in front of the OCTA and rests his head and chin on support. The algorithm used was split-spectrum amplitude-decorrelation angiography (SSADA), A-scan rate of the instrument was 70000 scans/s, the light source was centered on 840 nm, the bandwidth was 45 μm, the axial resolution obtained was approximately 5 μm, the beam width was 22 μm, and the number of A-scans required to reconstruct each B-scan was 216 A-scans. The number of B-scans at each fixed position was five consecutive B-scans, the number of locations along the slow transverse direction to form a 3D data cube was 216 locations, the B-scan frame rate per second was 270 frames, and acquired raster scans were four volumetric raster scans (two horizontal and two vertical). An area of 6 × 6 was centered on the fovea and was scanned. The AngioVue system included an orthogonal registration algorithm called motion correlation technology which minimizes motion artifacts produced by involuntary saccades and changes in fixation during data acquisition. The combination of motion-corrected OCTA along with the corresponding OCT intensity en face image and OCT B-scans allows a direct comparison of OCTA’s structural and functional information. Automatic segmentation of intraretinal layers was: superficial capillary plexus (SCP), 3 μm below inner limiting membrane to 15 μm below inner plexiform layer; deep capillary plexus (DCP) 15 to 70 μm below IPL; outer retina 70 μm below IPL to 30 μm below retinal pigment epithelium reference; and choroidal capillary, 30 μm to 60 μm below RPE reference. A scan size of 6 × 6 mm was used. The following quantitative data were obtained: vessel density perfusion percentage (VDP) of superficial capillary plexus (SCP) and deep capillary plexus (DCP) for the whole image, perifoveal and parafoveal areas; thickness of SCP and DCP for the whole image, parafoveal and perifoveal area; FAZ and central macular thickness (CMT). The following qualitative data were obtained: capillary non-perfusion, disorganization of vessels in SCP and DCP, and the presence of microaneurysms. There was 1 to 10 score for scan quality assessment that was automatically calculated. All eyes examined in our study had ≥ a score of 5. No eyes were excluded from the study.

### Statistical analysis

Data were processed using Statistical Package for Social Sciences (SPSS) version 26. Numerical data were summarized as means and standard deviations. Categorical data were presented as numbers and percentages. Frequency was calculated as percentages. Chi-square or Fisher’s tests were used to compare categorical data. Kruskal–Wallis test and Mann–Whitney test were used for non-normally distributed quantitative variables.

To determine the independent effect of different factors on the presence of retinopathy, factors which had significance level less than 0.10 were selected to enter into stepwise logistic regression analysis. Logistic regression was done to give adjusted odds ratio and magnitude of the effect of different risk factors in relation to retinopathy. Odds ratio (OR) and 95% confidence interval (95% CI) were done and 95% CI that does not contain 1.0 is considered significant.

Receiver operating characteristic (ROC) curve was done to determine the best cutoff point, sensitivity, specificity, and area under the curve. Accuracy was assessed based on the area under the ROC curve. All tests were two-tailed, and *P* value ≤ 0.05 is considered significant.

## Results

### Clinical and laboratory characteristics of subjects with prediabetes and normal control

One hundred and thirty-two subjects participated in this study. The prediabetes group included 66 patients and the normal control group included 66 individuals. The mean age of prediabetic individuals was 42 years with no statistically significant difference in age between the two groups (*P* = 0.084). Most of the subjects with prediabetes were females (83.3%). Individuals with prediabetes had significantly higher BMI (35.1 ± 6.4 vs. 22.1 ± 2.2; *P* < 0.001), and HbA1c (5.9 ± 0.4 vs. 4.8 ± 0.5; *P* < 0.001) compared to normal subjects. About 12% of individuals with prediabetic states had microalbuminuria. Retinopathy was found in 36.4% of prediabetic subjects by OCTA but only in 10.6% by fundus photography. Retinopathy among the prediabetes group was only in the form of mild non-proliferative diabetic retinopathy (Table 1).

**Table 1:** Qualitative and quantitative data measured by OCTA in prediabetes and normal groups

Variable		Prediabetes group *N* (%)	Normal group *N* (%)	*P* value
*Qualitative data measured by OCTA*				
Area of capillary non-perfusion	Yes	71 (53.8)	7 (5.3)	< 0.001
	No	61 (46.2)	125 (94.7)	
Disorganization of SCP vessels	Yes	75 (56.8)	34 (25.8)	< 0.001
	No	57 (43.2)	98 (74.2)	
Disorganization of DCP vessels	Yes	26 (19.7)	15(11.4)	0.062
	No	106 (80.3)	117 (88.6)	

Variable	Prediabetes group Mean ± SD	Normal group Mean ± SD	*P* value
*Quantitative data measured by OCTA*			
Foveal avascular zone (mm^2^)	0.32 ± 0.14	0.31 ± 0.09	0.347
Vessel density perfusion percentage of SCP—whole image (%)	49.6 ± 4.6	53 ± 3.1	< 0.001
Thickness of SCP -whole image (μm)	276.7 ± 15.1	277.2 ± 13.7	0.912
Vessel density perfusion percentage of SCP—parafoveal area (%)	51.3 ± 5.9	54.1 ± 3.4	< 0.001
Thickness of SCP—parafoveal area(μm)	314.1 ± 14.8	315.1 ± 11.7	0.745
Vessel density perfusion percentage of SCP—perifoveal area (%)	49.9 ± 4.6	52.9 ± 2.6	< 0.001
Thickness of SCP—perifoveal area(μm)	274.8 ± 15.7	273.2 ± 22.6	0.524
Vessel density perfusion percentage of DCP-whole image (%)	49.7 ± 7.5	55.1 ± 4.4	< 0.001
Thickness of DCP -whole image(μm)	276.7 ± 15.1	277.2 ± 13.7	0.912
Vessel density perfusion percentage of DCP- parafoveal area(%^2^)	54.6 ± 6.6	58 ± 4.2	< 0.001
Thickness of DCP parafoveal area(μm)	314.1 ± 14.8	314.9 ± 12.3	0.617
Vessel density perfusion percentage of DCP- perifoveal area (%)	51.2 ± 8	55.4 ± 4.4	< 0.001
Thickness of DCP perifoveal area(μm)	274.8 ± 15.7	273.2 ± 22.6	0.524
Central Macular thickness(μm)	241.2 ± 21.6	244.4 ± 18.2	0.200

^a^*N* number of eyes and percentages; *SCP* superficial capillary plexus; *DCP* deep capillary plexus. *P* value < 0.05 is considered significant

### Comparison of OCTA variables between subjects with prediabetes and normal control

**Fig. 1:**
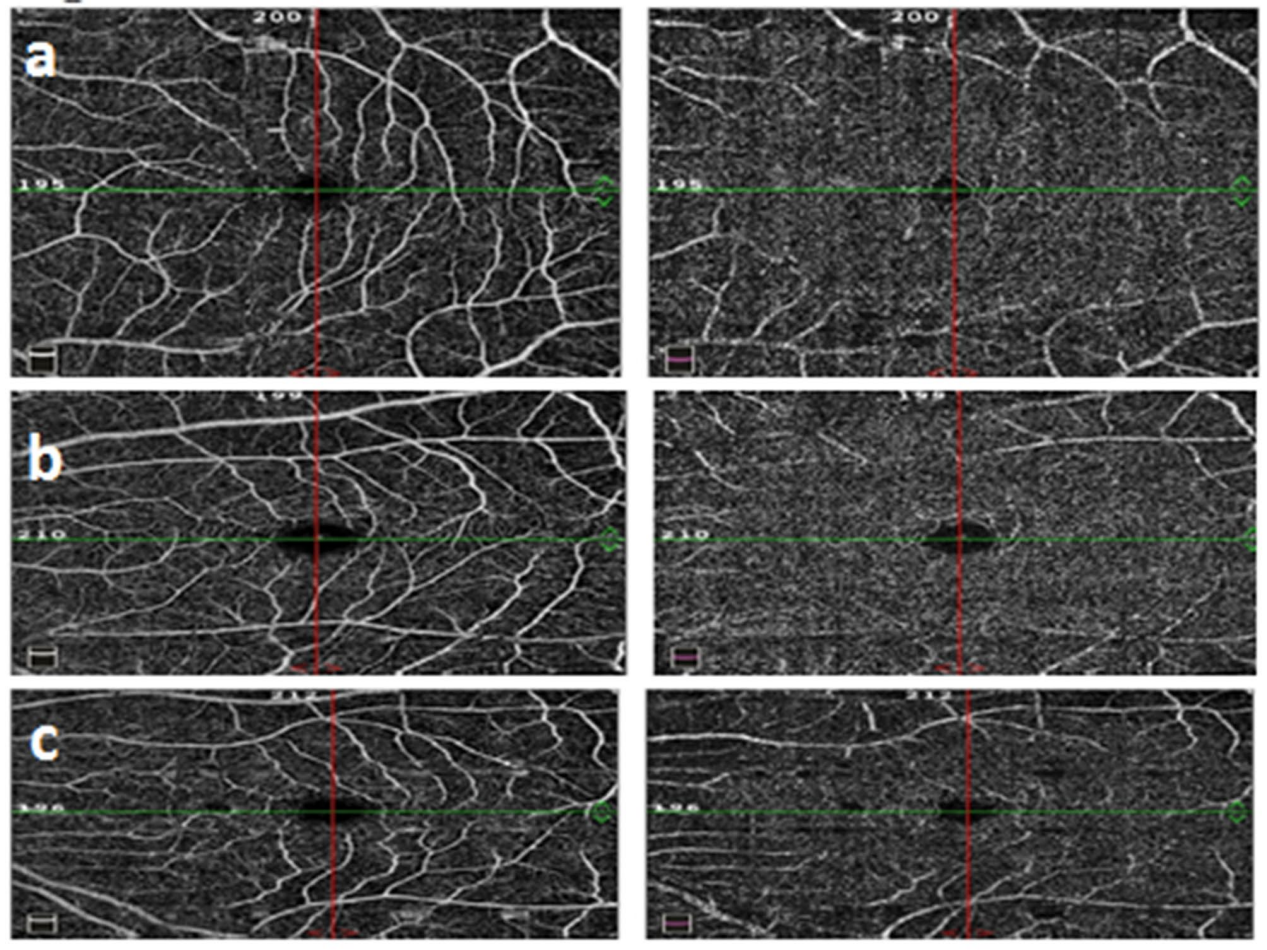
Comparison of OCTA in normal control, prediabetics without retinopathy and prediabetics with retinopathy. Optovue images with volumetric scan 6 × 6 mm^2^ centered on the maculae. It shows reduced VPD of SCP (1st column) and DCP (2nd column) in prediabetics without DR (**b**), and prediabetics with DR(**c**) compared to control (**a**). Prediabetics with DR (**c**) had lower VPD of DCP compared to prediabetics without DR (**b**)

Areas of capillary non-perfusion were detected in 53.8% of the prediabetes group and 5.3% of the normal group (*P* < 0.001). Disorganization of SCP vessels was more frequent in the prediabetes group compared to the control (*P* < 0.001); VDP of SCP and DCP of the whole image, parafoveal area, and perifoveal area was significantly lower in the prediabetes group compared to the control (*P* < 0.001, for all) (Table 1) (Fig. 1a).

### Clinical and laboratory characteristics of the prediabetic subjects with and without retinopathy

Based on the presence or absence of microaneurysms, prediabetic individuals were divided into two groups: a group with retinopathy included 40 eyes and a group without retinopathy included 102 eyes. There was no statistically significant difference between prediabetic subjects with retinopathy and without retinopathy regarding age (*P* = 0.104), gender (*P* = 0.309), BMI (*P* = 0.542), HbA1c (*P* = 0.938), and microalbuminuria (*P* = 0.076).

### Comparison of OCTA results of the prediabetic subjects with and without retinopathy

The presence of areas of capillary non-perfusion, disorganization of SCP vessels, and disorganization of DCP vessels were more frequent in the prediabetics with retinopathy compared to those without retinopathy (*P* = 0.002; *P* = 0.022; *P* =  < 0.001, respectively). VDP of DCP of the whole image, parafoveal area, and perifoveal area was significantly lower in prediabetes group with DR compared to those without DR (*P* < 0.001, for all). However, there was no significant difference between the prediabetics with and without retinopathy regarding VDP of SCP of the whole image (*P* = 0.562), parafoveal area (*P* = 0.279), and perifoveal area (*P* = 0.841) (Table 2) (Fig. 1b and c).

**Table 2:** Qualitative and quantitative data measured by OCTA in prediabetes subjects with and without retinopathy

Variable		Prediabetes with retinopathy *N* (%)	Prediabetes without retinopathy *N* (%)	*P* value
Qualitative data measured by OCTA				
Area of capillary non-perfusion	Yes	30(75)	41(44.6)	0.002
	No	10(25)	51(55.4)	
Disorganization of SCP vessels	Yes	29(72.5)	46(50)	0.022
	No	11(27.5)	46(50)	
Disorganization of DCP vessels	Yes	16(40)	10(10.9)	< 0.001
	No	24(60)	82(89.1)	

Variable	Prediabetes group Mean ± SD	Normal group Mean ± SD	*P* value
Quantitative data measured by OCTA			
Foveal avascular zone (mm^2^)	0.3 ± 0.13	0.33 ± 0.15	0.247
Vessel density perfusion percentage of SCP—whole image (%)	49.9 ± 4.2	49.4 ± 4.8	0.562
Thickness of SCP—whole image (μm)	275.6 ± 14.9	277.2 ± 15.2	0.586
Vessel density perfusion percentage of SCP—parafoveal area (%)	50.5 ± 6.2	51.7 ± 5.8	0.279
Thickness of SCP—parafoveal area(μm)	310.6 ± 14.5	315.6 ± 14.8	0.078
Vessel density perfusion percentage of SCP—perifoveal area (%)	49.8 ± 4.4	50 ± 4.7	0.841
Thickness of SCP—perifoveal area(μm)	273.3 ± 15.3	275.4 ± 16	0.473
Vessel density perfusion percentage of DCP-whole image (%)	46.3 ± 7.6	51.2 ± 7	< 0.001
Thickness of DCP -whole image(μm)	275.6 ± 14.9	277.2 ± 15.2	0.586
Vessel density perfusion percentage of DCP- parafoveal area(%^2^)	51.6 ± 6.3	56 ± 6.2	< 0.001
Thickness of DCP parafoveal area(μm)	310.6 ± 14.5	315.6 ± 14.8	0.078
Vessel density perfusion percentage of DCP- perifoveal area (%)	47.4 ± 8.3	52.8 ± 7.3	< 0.001
Thickness of DCP perifoveal area(μm)	273.3 ± 15.3	275.4 ± 16	0.473
Central Macular thickness(μm)	245.2 ± 29	239.4 ± 17.3	0.251

*N* number of eyes. *SCP* superficial capillary plexus; *DCP* deep capillary plexus. *P* value < 0.05 is considered significant

### Multivariate analysis of factors associated with retinopathy in subjects with prediabetes

Multivariate analysis assessing factors independently associated with retinopathy in prediabetics was performed. Variables that had a significance level less than 0.100 were selected to enter into stepwise logistic regression analysis. Variables entered into the model were: age, VDP of DCP of the whole image, parafoveal area, and perifoveal area, area of capillary non-perfusion, disorganization of SCP, and disorganization of DCP. VDP percentage of DCP of perifoveal area (*β* coefficient: − 0.10, OR: 0.91, 95% CI: 0.86–0.96, *P* < 0.001) and disorganization of DCP (*β* coefficient: 1.93, OR: 6.89, 95% CI: 2.5–18.8, *P* < 0.001) were the most important predictors for retinopathy in subjects with prediabetes. For every unit decrease in VDP of DCP of the perifoveal area, there is a 10% increased risk of retinopathy. Patients with disorganization of DCP have nearly seven times increased risk of retinopathy.

## Diagnostic performance of VDP of DCP areas as predictors of retinopathy in subjects with prediabetes

The AUC of VDP of DCP of the whole image was 0.80 (95% CI: 0.75–0.85; *P* < 0.001), the parafoveal area was 0.77 (95% CI: 0.72–0.82; *P* = 0.001), and the perifoveal area was 0.77 (95% CI: 0.72–0.82; *P* < 0.001). The sensitivity of VDP of DCP of parafoveal area less than 52.5%, perifoveal area less than 45.4%, and whole area less than 45.8% was 71.4%, 64.3%, and 64.3%, respectively, for prediction of retinopathy in prediabetic state. VDP of DCP of the perifoveal area had the highest specificity (91.2%), followed by VDP of DCP of the whole image (90%) and lastly VDP percentage of DCP of the parafoveal area (85.6%) for prediction of retinopathy in subjects with prediabetes.

## Discussion

In the current study, signs of DR were detected in 36.4% of patients with prediabetes, while fundus photography detected DR only in 10.6% of them. Similar results were obtained by Yang et al. in patients with type 2 DM who were found to have retinal microvascular changes in 40% of them by OCTA, but no DR findings were detected by ophthalmoscopy [18]. OCTA is a promising marker for early DR because it can produce a dye-free image that can detect capillary-level microangiopathy which cannot be seen by fundus photography. However, some studies disagree, arguing that clinical examination and glycemic control should continue to be the main clinical criterion during DR screening and that OCTA is not the best technique for identifying preclinical alterations in diabetic patients [19]. ﻿﻿In ﻿﻿contrast, our results showed no significant association between common risk factors of retinopathy including age, sex, BMI, HbA1c, and ACR, and the presence of DR in people with prediabetes [20]. This is not exceptional; as such, finding has been reported in a previous study by Chen et al. [21] that no significant correlation was found between DR and age, BMI, creatinine, ACR, and lipid profile in prediabetics. Moreover, we found significant changes in prediabetic eyes detected by OCTA compared to healthy people suggesting its usefulness in the prediction of early DR in the prediabetes stage .

Non-perfusion regions are black, flow-void patches on OCTA that are bordered by broken capillaries [9]. The non-perfused areas around the macula in diabetic eyes were studied previously using OCTA and have shown an increase in these areas [9]. Ishibazawa et al. [10] found that non-perfusion areas were much larger in SCP than in the DCP. Another study measured the percent area of non-perfusion and found extensive progression in this area with increasing severity of DR [22]. Furthermore, one study reported capillary non-perfusion in patients with DM without evidence of DR [23]. In our study, the eyes of people with prediabetes had higher capillary non-perfusion areas than the eyes of normal control; also, prediabetes eyes with DR had higher capillary non-perfusion areas than prediabetes eyes without DR. These results suggest the role of inadequate retinal blood flow in the pathogenesis of DR in the prediabetes state.

Many previous studies showed a significant reduction in vessel density and perfusion density in the macular region in diabetic patients with retinopathy compared to healthy controls [24, 25]. However, reduced perfusion density has shown debatable results in patients with diabetes without DR. Some studies documented the reduction of perfusion density only in patients with DR, with no significant changes between non-diabetic and diabetic subjects without DR [26, 27]. In contrast, other studies showed a considerable decrease in the perfusion density in patients with diabetes without clinical manifestations of DR [27–29]. Only a few studies have been conducted on subjects with prediabetes. Xu et al. [30] reported a decrease in vessel area density and a widening of the mean vessel density within the SCP and DCP in prediabetes eyes . Similarly, we found a reduction in VDP in both SCP and DCP in prediabetes eyes compared to normal control. However, we found a reduction in VDP in DCP but not in SCP of the whole image, parafoveal area, and perifoveal area in prediabetes eyes with DR compared to those without DR.

VDP of SCP and DCP in diabetic eyes has been analyzed in previous studies. Some research showed a reduction in vessel density perfusion in both SCP and DCP in patients with DR [31]. However, many studies pointed to the significant correlation between the VDP of DCP and DR [32–34]. Even in diabetic patients without signs of DR, there was a significant reduction in VDP in DCP without a significant difference in the vessel density of SCP [35–38]. Furthermore, a reduction in vessel density was found to occur more rapidly in the DCP than in the SCP during the progression of DR [9]. Our results deduced that the VDP of DCP-whole area < 45.8, VDP of DCP parafoveal < 5 2.5, and VDP of DCP perifoveal < 45.4 are good markers of DR making DCP changes at most important in the prediction of DR in prediabetes subject. The difference in the perfusion pressure between the SCP and the DCP could illuminate the more prominent vascular changes in the DCP in DR [39]. Furthermore, previous investigations demonstrated higher susceptibility of the deep foveal plexus to endothelial damage [40].

With the progression of DR, FAZ increases due to damage of the surrounding capillary [41]. OCTA is more sensitive than fundus photography in the distinction of the central and parafoveal macular microvasculature, particularly for FAZ disruption, as reported by Soares et al. [42]. Although some previous studies in patients with DM showed that FAZ measurements deviate noticeably from controls, there was no difference between control group and prediabetic group regarding FAZ in our study. This is consistent with what was previously described in patients with diabetes without DR [25, 35]. Similarly, Airas et al. [15] reported no differences in FAZ area between prediabetic patients and non-diabetic controls. Furthermore, VDP was found to be a more sensitive sign in differentiation between healthy individuals and DM patients than FAZ measurement [43]. A preserved FAZ morphology with a decreased VDP in prediabetics could be explained by that the early pathological changes in prediabetic patients are mild and no typical lesions DR are found. To the best of the author's knowledge, only one study evaluated OCTA in the detection of retinopathy in a prediabetes state. Besides, our study compared the OCTA findings in prediabetes subjects with and without macular vascular changes showing significant disorganization and changes in VDP in DCP. These results highlight that macular changes could serve as biomarkers of microvascular injury at the prediabetes stage. This might have important clinical implications since the visualization and direct assessment of retinal microvascular changes could be an indication for screening and early intervention of other target organ damage in patients with prediabetes. However, this study has some limitations. First, it was a cross-sectional study so the duration of impaired glucose metabolism cannot be assessed therefore; a longitudinal cohort study in future may provide more evidence. Second, all of the study populations were Egyptians so the applicability of our results on other ethnicities is questionable. Third, it was preferred to have a larger sample size. Fourth, OCTA as a novel technique is subject to some limitations, including increased costs, longer acquisition times, and low reproducibility, often bearing difficulties in image acquisition and artifacts. For example, impaired blood flow may lead to "flow-void" images without necessarily corresponding to non-perfusion. However, in the current study, we applied strict exclusion criteria, and all eyes examined in our study had a scan quality score ≥ 5.

## Conclusions

Retinal microvascular complications can evolve earlier than the diagnosis of overt diabetes, which makes scanning for DR in subjects with prediabetes mandatory for its prevention or delay. OCTA could be a promising tool in the early detection of DR in the prediabetes stage with vessel disorganization of DCP and reduction of VDP of the DCP being early signs of DR in patients with prediabetes.

## Data Availability

The datasets generated during and/or analyzed during the current study are available from the corresponding author on reasonable request.
